# Acoustic transmission loss in Hilbert fractal metamaterials

**DOI:** 10.1038/s41598-023-43646-1

**Published:** 2023-11-04

**Authors:** Gianni Comandini, Morvan Ouisse, Valeska P. Ting, Fabrizio Scarpa

**Affiliations:** 1 Bristol Composite Institute (BCI), School of Civil, Aerospace and Mechanical Engineering (CAME), University of Bristol, Bristol, UK; 2 SUPMICROTECH, Université de Franche-Comté, CNRS, Institut FEMTO-ST, 25000 Besançon, France; 3 Research School of Chemistry, Australian National University, Canberra, ACT 2601 Australia

**Keywords:** Aerospace engineering, Civil engineering, Mechanical engineering, Engineering, Materials science

## Abstract

Acoustic metamaterials are increasingly being considered as a viable technology for sound insulation. Fractal patterns constitute a potentially groundbreaking architecture for acoustic metamaterials. We describe in this work the behaviour of the transmission loss of Hilbert fractal metamaterials used for sound control purposes. The transmission loss of 3D printed metamaterials with Hilbert fractal patterns related to configurations from the zeroth to the fourth order is investigated here using impedance tube tests and Finite Element models. We evaluate, in particular, the impact of the equivalent porosity and the relative size of the cavity of the fractal pattern versus the overall dimensions of the metamaterial unit. We also provide an analytical formulation that relates the acoustic cavity resonances in the fractal patterns and the frequencies associated with the maxima of the transmission losses, providing opportunities to tune the sound insulation properties through control of the fractal architecture.

## Introduction

Excessive noise can significantly impact human health and lead to serious pathology^1,2^. Traditional noise transmission mitigation strategies^3,4^ have followed mass law principles^5^, with a resulting drawback in terms of high mass and thickness of the materials used^6^. Mass-spring-mass models exploit other mechanisms, with the development of designs based on one or multiple coupled degrees of freedom and combinations of materials^7^. Spring-mass models are typically used in acoustic architecture to separate environments with rational use of the space available. Other more recent but consolidated technologies exploit the use of porous materials as perforated^8–10^ and/or microperforated panels^11^, or through thermo-viscous acoustic energy dissipation in the internal porous skeletons of foams with various internal geometry and materials compositions^12–16^. Thermo-viscous energy dissipation technologies also theoretically allow acoustic dampening of any frequency, with the only limitation represented by the available room for the porous liners. Because of space constraint limitations, porous materials are generally adopted to mitigate noise within the middle and high-frequency ranges. Another technique to obtain a desired transmission loss (TL) for a predetermined interval of frequencies is by using Helmholtz resonators^17–20^. However, resonators are only efficient for narrow sets of frequency ranges and may involve the use of considerable space for the spring part of the Helmholtz resonator for low frequencies applications^21,22^. More recent techniques for noise management are promising because of their lightweight characteristics and their use of different mechanisms to achieve sound absorption and transmission loss^16,23–30^. Metamaterials for noise control are moving from conceptual design^31–34^ to practical implementation^35–42^. Fractal geometries have been used to design electromagnetic and mechanical metamaterials^43–46^. Moreover, fractal mechanical metamaterials possess strength, shape stability under large deformations, and significant damage tolerance^47^. The Hilbert lattice^48^ is one of the most heavily explored fractals in metamaterial design, especially for electronics/electromagnetic applications. Fractal antennas have shown enhanced performance compared to traditional designs since they can operate at multiple frequencies without load and are more compact than those featuring other geometries^49–52^. Recent studies in the field of acoustics have improved the understanding of the Hilbert space-filling curve pattern in metamaterials, providing numerous benefits ranging from high porosity, multiple resonances, multi-modes, and sub-wavelength scales^53,54^. This work describes, for the first time, a numerical and experimental evaluation of the acoustic transmission loss capability of Hilbert fractal metamaterials.

A point of particular novelty here is in the improved understanding of the physics behind the frequencies corresponding to transmission loss peaks. As will be demonstrated, those frequencies correspond to the phenomenon of impedance mismatch, with an analytical formulation that describes how to calculate those frequencies also being presented. Furthermore, we demonstrate that the use of the Hilbert fractal pattern to design passive tools for acoustic control relies on the capability of the metamaterials geometries to provide tunable transmission loss in the lower part of the acoustic spectrum. This is an advantage when compared with commonly used acoustic porous materials having the same thickness^55,56^ or - more significantly - to the narrow-band effect of Helmholtz resonators^57,58^ or straight slits with similar or narrower widths (see the Supplementary Information).

**Figure 1:**
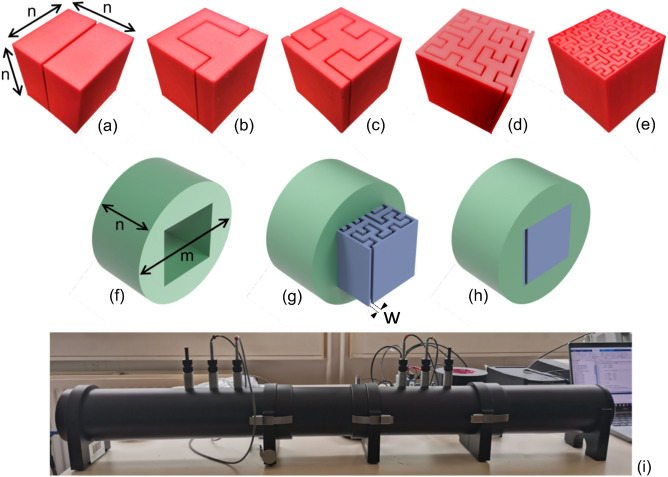
The Hilbert fractal acoustic metamaterials and the impedance tube used in this work. (**a**) The 0th order with underlined *n*, the side dimension of the cube − 50.8 mm, in this case. (**b**) 1st, (**c**) 2nd, (**d**) 3rd, and (**e**) 4th orders of the Hilbert fractal. (**f**) Sample holder used for the samples inside the test room of the impedance tube. The holder has an external diameter *m* of 100 mm and thickness *n* of 50.8 mm. (**g**) Example of the cubic specimen (**d**), having been partially inserted into the holder (**f**), showing the gap width *w*. (**h**) Holder and sample completely assembled. (**i**) The impedance tube.

The fractal topologies covered here range from the 0th (Fig. 1a) to the 4th order (Fig. 1e). We have used additive manufacturing to generate samples of fractal metamaterials made using polylactic acid (PLA) because of its recyclability and good printability. One of the design features of the fractal specimens is the presence of inlets and outlets for the impinging and exiting acoustic waves (Fig. 1a–e). The geometry of the fractal patterns is here defined in nondimensional terms, using the lateral dimension of the metamaterial sample * n* (50.8 mm in our case), the gap width *w* (see Fig. 1g), and the fractal order $\xi$. The parameter $\eta ={{\textsf {\textit{w}}}}/{{\textsf {\textit{n}}}}$ represents the ratio between gap width $({{\textsf {\textit{w}}}})$ and side dimension of the specimen $({{\textsf {\textit{n}}}})$; meanwhile, the porosity is defined as $\varphi =\eta (2^{\xi })$.

An impedance tube with four microphones was used to obtain the experimental measurements of the TL (Fig. 1i). Numerical Finite Element simulations using COMSOL Multiphysics^59^ featuring different models representing the air inside the cavities of the fractal metamaterials, and analytical models were also performed. The models were used here to benchmark the results and understand some of the physical mechanisms underpinning the amplitude and frequency of the TL peaks.

**Figure 2:**
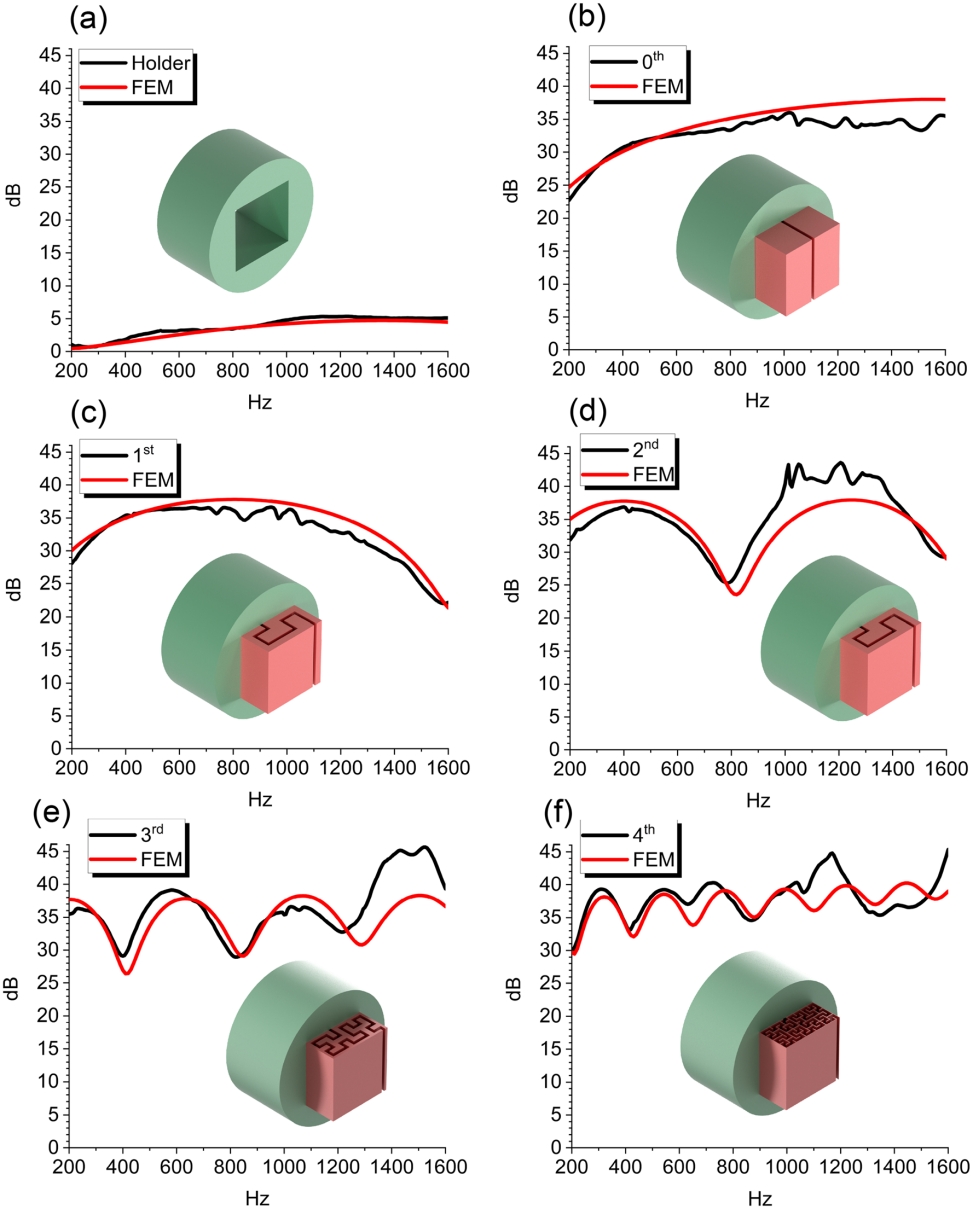
Comparison of the transmission loss between experimental results (black) and FEM (red) for the various Hilbert fractal orders. All the measurements and simulations were made taking into account the cylindrical holder of Fig. 1f. (**a**) Only the holder (Fig. 1f). (**b**) Zeroth (Fig. 1a). (**c**) First (Fig. 1b). (**d**) Second (Fig. 1c). (**e**) Third (Fig. 1d). (**f**) Fourth (Fig. 1e) orders.

## Results and discussion

We performed a comparison between experimental and finite element-simulated transmission losses for the Hilbert fractal metamaterials with a gap width of 1.0 mm (i.e., corresponding to a value of $\eta$ equal to 2 %). The size of the gap is the same on both sides of the sample. The finite element results here are related to narrow-acoustics models of the cavity^59^ inside the fractal metamaterials. There is a good agreement between experiments and simulations, as can be seen in Fig. 2. As also shown in the Supplementary Information (Fig. S2), Hilbert geometries from the second order with ${{\textsf {\textit{w}}}}=1$ mm can provide a greater transmission loss compared to a block of bulk PLA with the same dimension *n*. The mechanism that generates the peaks of the transmission loss is an impedance mismatch between the air inside the tube and the metamaterial.

The metamaterial provides an increasing number of minimum transmission coefficient (TC) values in the frequency spectrum investigated according to the fractal order, whereas the peaks of the transmission loss correspond to frequencies related to the minimum TC values. The transmission loss has a behaviour similar to an inverse pass filter, for which the transmission loss is maximum and therefore the TC is at its minimum. Moreover, as shown in Fig. S4, the high values of the peak of the reflection coefficient highlight the multi-resonance behaviour due to the Fabry-Pérot resonance. The sample made of bulk PLA has its own Fabry-Pérot resonant frequencies. Those however lie outside the spectrum of frequencies investigated in this work.

At the same time, the metamaterial can minimise and maximise the mismatch at multiple frequencies, as shown in Fig. S4. A dip in the reflection coefficient corresponds to a drop in the transmission loss output. The fractal acoustic metamaterial can therefore provide multiple maximisations and minimisations of the impedance mismatch by a single fractal geometry cavity.

**Figure 3:**
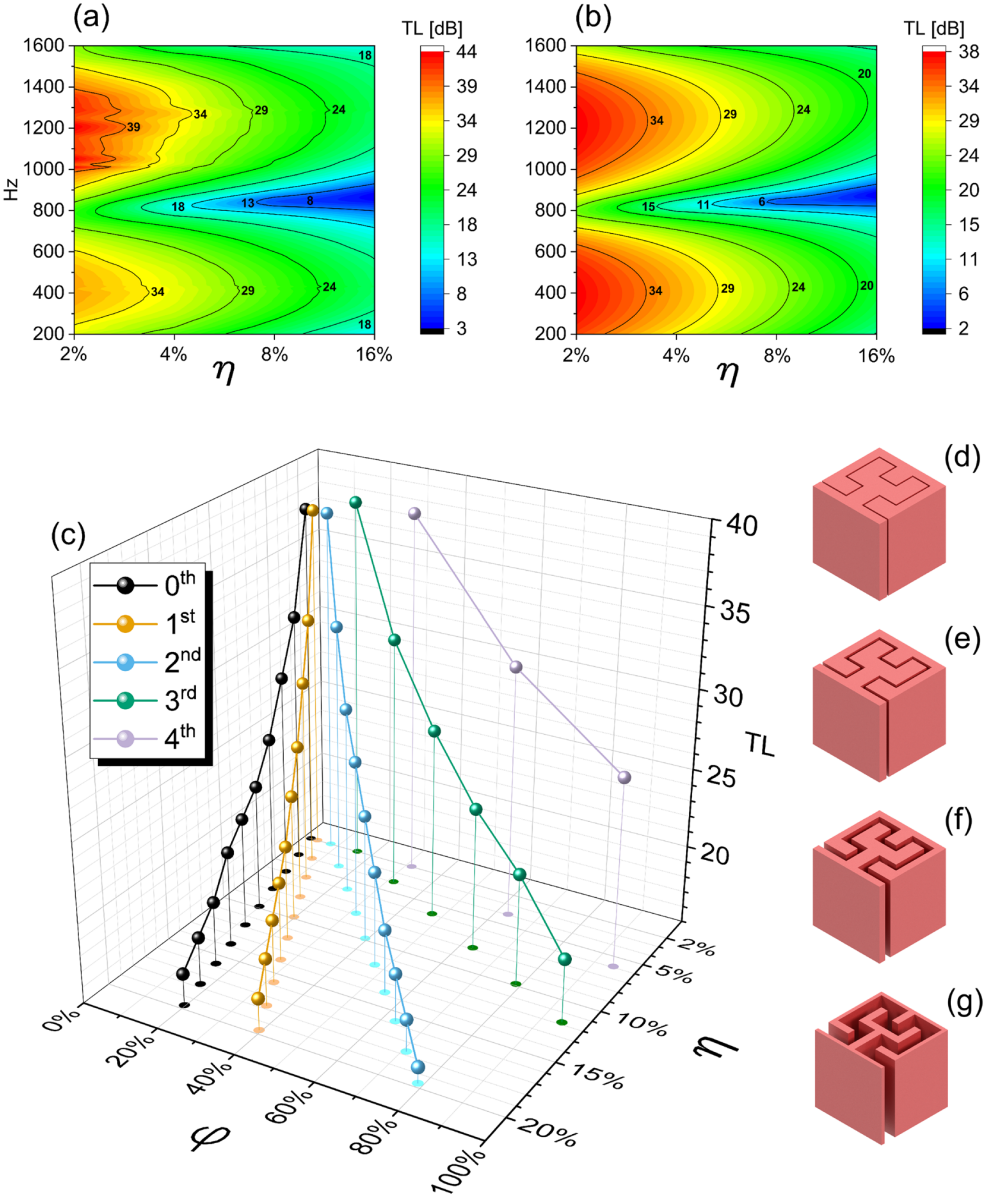
Experimental (**a**) and FE (**b**) contour plots of the TL as a function of the frequencies and normalised gap widths. (**c**) Variation of the transmission loss peaks calculated via FEM for the fractal orders and their porosity when the normalised gap changes. Example of the 2nd order Hilbert fractal with a gap width of (**d**) 1 mm, (**e**) 2 mm, (**f**) 4 mm, and (**g**) 8 mm.

A description of the model, and a comparison between lossless, viscous and experimental results related to configurations from the 0th to the 4th fractal order are presented in the Supplementary Information. Both viscous-based and lossless formulations for the fluid are able to capture the overall transmission loss properties inside the fractal cavities, and the narrow-acoustics simplification is adequate to follow the experimental results; moreover, as described by the work of Ward, et al^60^, the particle velocity field inside the fractal channels is lower when thermoviscous losses are considered in the FEM model, as shown in the Supplementary Information Fig. S5. The lossless model however provides large dips of transmission loss at frequencies slightly larger than those predicted by the viscous models, and provided by the experimental results. It is interesting to note the behaviour of the TL for the various configurations. The order zero (i.e., the topology with the cavity extending to the full length of the cubic sample - Fig. 1a). has a peak of the transmission loss equal to 37 dB at 1546 Hz (Fig. 2b). A peak of 37 dB is however observed at 785 Hz in the case of the first fractal order (Fig. 2c). The second Hilbert fractal order possesses two peaks at 390 Hz and 1212 Hz, both with TL values of 37 dB and 38 dB, respectively (Fig. 2d). The third order shows the presence of four transmission loss peaks at 195 Hz, 611 Hz, 1033 Hz, and 1455 Hz, all with a TL value of 38 dB (Fig. 2e). Finally, the fourth fractal order exhibits seven TL peaks at 310 Hz, 526 Hz, 743 Hz, 961 Hz, 1180 Hz, 1399 Hz, and 1618 Hz, with TL values of 38 dB, 38 dB, 39 dB, 39 dB, 40 dB, 40 dB, and 41 dB, respectively (Fig. 2f). We also include the results related to the transmission losses of the empty holder (Fig. 1f); as expected, the TL values here were the lowest amongst all the configurations considered (Fig. 2a). In summary, both experiments and simulations show the presence of multiple peaks and minima of the transmission loss; the number of those peaks and dips depends on the fractal order of the pattern of the metamaterial considered. The maxima of the transmission loss all show a nearly constant magnitude, with their position dependent on the length of the fractal and, consequently, on a higher Hausdorff dimension^61^. Despite the complexity of the acoustic behaviour of these fractal-shaped metamaterials, it is possible to identify some general trends in their TL response. Increased fractal orders feature larger numbers of TL peaks with a very similar magnitude (see Table 2 and Fig. 3c)). This behaviour can be explained by observing that the performance of the TL magnitude of the metamaterial is mainly dependent upon the geometry of the opening slot, rather than on the fractal order itself.

**Table 1:** Normalized gap, fractal orders, porosity, and TL values for the classes of fractal metamaterials evaluated in this work. The leftmost column shows the normalized gap values, while the second column displays the corresponding fractal orders, which are proportional to the gap width. The third column contains the equivalent porosity of each fractal order in terms of relative gap width. The fourth column shows the values of the peaks of the transmission loss calculated via FEM and related to the first cavity mode of the fractals for a given gap width.

Gap width $\eta$ (%)	Fractal order $\xi$	Porosity $\varphi$ (%)	TL [dB]
2	0th, 1st, 2nd, 3rd, 4th	2, 4, 8, 16, 31	37
4	0th, 1st, 2nd, 3rd, 4th	4, 8, 16, 31, 63	31
6	0th, 1st, 2nd, 3rd, 4th	6, 12, 24, 47, 94	27
8	0th, 1st, 2nd, 3rd	8, 16, 31, 63	25
10	0th, 1st, 2nd, 3rd	10, 20, 39, 79	23
12	0th, 1st, 2nd, 3rd	12, 24, 47, 94	21
14	0th, 1st, 2nd	14, 28, 55	19
16	0th, 1st, 2nd	16, 31, 63	18
18	0th, 1st, 2nd	18, 35, 71	17
20	0th, 1st, 2nd	20, 39, 79	16

**Table 2:** Cavity resonances predicted via Finite Element (narrow viscous model) and the analytical formulation for the various fractal orders. The Finite Element results related to each mode are calculated by averaging the values of those modes over the different gap widths considered in this work. The standard deviations of those frequencies are normalised against the corresponding average value. The last column refers to the analytical resonance frequency related to an open-closed cavity..

Fractal order $\xi$	Mode number	FEM mean [Hz]	Normalised standard deviation (%)	Eq. (1)
0th	1st	1472.2	1.6	1548.7
1st	1st	811.2	0.3	807.3
2nd	1st	426.6	0.8	412.6
2nd	2nd	1295.3	1.3	1237.7
3rd	1st	224.8	0.8	208.6
3rd	2nd	685.3	1.1	625.8
3rd	3rd	1148	1.5	1043
3rd	4th	1602.5	1.3	1460.2
4th	1st	328	0.8	314.7
4th	2nd	553.7	0.7	524.5
4th	3rd	781	1.1	734.3
4th	4th	1008	1.1	944.1
4th	5th	1235.3	1.0	1153.9
4th	6th	1462.7	1.2	1363.7
4th	7th	1689.3	1.3	1573.5

Table 1 shows the dependence of the magnitude of the transmission loss peak values from the FEM simulations versus the normalised gap width, the fractal orders and the porosity of the metamaterials. One can notice that the most significant factor affecting the values of the transmission losses is the gap width, regardless of the fractal order or porosity considered (see also Fig. 3c). Of particular interest is also the effect of the fractal orders on the frequencies corresponding to the peak of the transmission losses, which are also known to affect the acoustic absorption properties of Hilbert fractal metamaterials^53^. The results suggest that while the order of the fractal pattern can impact both peak TL magnitudes and related frequencies, it is not the dominant factor to control those frequencies in configurations with varying normalised gap widths. Furthermore, the results indicate that variations of the porosity have a limited effect on the transmission losses (Fig. 3c and Table 1).

The results from the experimental and numerical transmission losses show that the frequencies corresponding to the peak of the TL can be approximated using equation (Eq. 1), which is also adopted for the design of musical (wind) instruments^62,63^. Formula (Eq. 1) is related to open-closed one-dimensional resonating cavities and considers even harmonics only (i.e., $r = m+1$ with $m\in \mathbb {N}$)^62^. The resonant frequency for an open-closed cavity representing the fractal path is equal to:

1 $$\begin{aligned} f_{n} = \frac{rc}{4(L+\Delta L)} \end{aligned}$$

In Eq. (1), *c* is the speed of sound in air, $343\ m/s$ and $L={{\textsf {\textit{ n}}}}(2^{\xi })$ is the length of the fractal cavity inside the metamaterial sample, and $\Delta L$ is the extra length of the fractal channel that needs to be considered due to thermoviscous losses^60,64^. The term $\Delta L$ is equal to $\frac{8{{\textsf {\textit{w}}}}}{3\pi }$ ^60^. A comparison of the normalised standard deviations of the of the frequencies related to peaks of the TL and calculated via FEM and the analytical formulas is shown in Fig. S6 of the Supplementary Information. The analytical formulas include the terms $\Delta L = 0$ (Fabry-Pérot model) and $\Delta L=\frac{8}{3\pi ^{\frac{3}{2}}}\sqrt{{{\textsf {\textit{nw}}}}}$ ^64^.

At the frequencies corresponding to the various cavity modes of the fractal patterns, the peak of the TL is always close to a value of 37 dB; this is true also within the low-frequency range within  250Hz–400Hz if the percentage opening on both sides of the metamaterial is $2\%$, no matter the fractal order considered (Table 1). This simplification is valid, especially for the case of the first peaks related to the first acoustic modes in the lower frequency range (Table 1). Table 2 summarises the numerical (narrow-acoustic approximation^59^) and analytical results related to the TL for different gap widths and fractal orders. The vibrational modes corresponding to the TL peaks are also indicated. The standard deviation of the distribution of frequencies associated with transmission loss peaks for the different gap widths is normalised against the value of 1688 Hz, which corresponds to the average frequency of the TL peak related to the 0th order when the gap width ($\eta$) is varied from 2% to 20% (Fig. 3d–g). Notably, the frequency corresponding to the TL peaks predicted by the analytical open-closed resonator formula is also close to the numerical FE one (Table  2, fifth column). The number of vibrational modes within the frequency range investigated here increases with the increasing fractal order, and the frequencies related to the TL peaks shift to higher values. The magnitudes of TL peaks are again almost constant, with only slight variations attributed to the change in the length of the fractal pattern inside the metamaterial. These findings imply that by increasing the fractal order, it is possible to achieve more resonant peaks, resulting in more effective broadband transmission loss, and also sound absorption^53^. Moreover, both numerical and analytical modelling approaches provide a good approximation of the actual behaviour of these fractal acoustic metamaterials (Figs. 2, and 3a,b).

## Conclusion

This work has shown the relationship between the fractal order and the transmission loss in acoustic metamaterials with Hilbert fractal patterns. The equivalent porosity and gap widths play a crucial role in determining the values of the transmission loss (Fig. 3c), with the gap width being the most significant factor impacting the magnitudes of the TL (Fig. 3a,b and Table 1) that create the impedance mismatch responsible for the generation of the TL maxima. Our experimental and numerical results show that a decrease in the transmission loss is generated by the increase of the gap width, with a consequent reduction of the performance of the acoustic metamaterial. We have also investigated the influence of fractal order on transmission loss. Our simulations show that the fractal order plays a role in determining the number of TL peaks, but not in their magnitude. Finally, the frequencies corresponding to the peaks of the transmission losses can be well approximated and predicted by considering the fractal patterns as open-closed one-dimensional cavities and calculating the corresponding resonance frequencies. The formula 1 makes it possible at the design stage to relate those frequencies to the fractal order and nondimensional gap width of the fractal acoustic metamaterial.

## Methods

All the samples were printed using the Raise3D Pro3 Plus 3D Printer. The machine was set up with a 0.1 mm layer height, 100% infill density, and an extrusion nozzle of 0.4 mm. 3DJake recycled PLA was used as the filament. The transmission loss have been measured following the ASTM E2611-09 standard^65^, using a Brüel & Kjær impedance tube (Fig. 1i) with a four microphones configuration and double load measurements. The gap widths of the metamaterials samples are 1 mm, 2 mm, 4 mm and 8 mm, corresponding to 2% (Fig. 3d), 4% (Fig. 3e), 8% (Fig. 3f) and 16% (Fig. 3g). All the tests were performed by mounting the fractal metamaterial sample in a cylindrical holder (Fig. 1f–h). The FEM simulations were performed using the Comsol Multiphysics 5.6 software^59^ with a 3D model of the impedance tube and the metamaterials specimens^53^. The FEM model does not consider the presence of the PLA material, only the fluid cavity is modelled, with loss-less, thermoviscous and narrow-viscous domains. The part of the impedance tube model outside the test room was represented with a lossless air fluid, with probes simulating the position of the four microphones. The frequency range investigated in both FEM and experiments was from 200 Hz to 1600 Hz.

### Supplementary Information

Supplementary Information.

## Data Availability

The datasets used and analysed during the current study are available from the corresponding author on reasonable request.
