# Supplementary material for: Acoustic transmission loss in Hilbert fractal metamaterials
Source: Sci Rep. 2023 Nov 4;13:19058. doi: 10.1038/s41598-023-43646-1 (PMC10625595; doi:10.1038/s41598-023-43646-1)
Supplement: Supplementary file 1 — Supplementary Information. [file 41598_2023_43646_MOESM1_ESM.pdf]

Supplementary Information

S.1 Comparison between experiments and FE models

Fig.S1 shows a comparison between experimental results and Finite Element Method (FEM) simulations for three different metamaterials configurations. The first FEM model (lossless) considers only the Helmholtz equations into account. The second FEM model (thermoviscous) accounts for the acoustic thermoviscous dissipation caused by the linearized Navier-Stokes equations. The third FEM model (narrow acoustics) takes into account the overall effects of the linearized Navier-Stokes equations, but at lower fidelity in narrow geometries. The Lossless model provides a good approximation of the experimental results in the case of the sample's holder (Fig.S1a), order zero (Fig.S1b) and the first Hilbert fractal order (Fig.S1c). However, the lossless modelling approach becomes less reliable in prediction of the TL values from the second fractal order onwards (Fig.S1d-f).

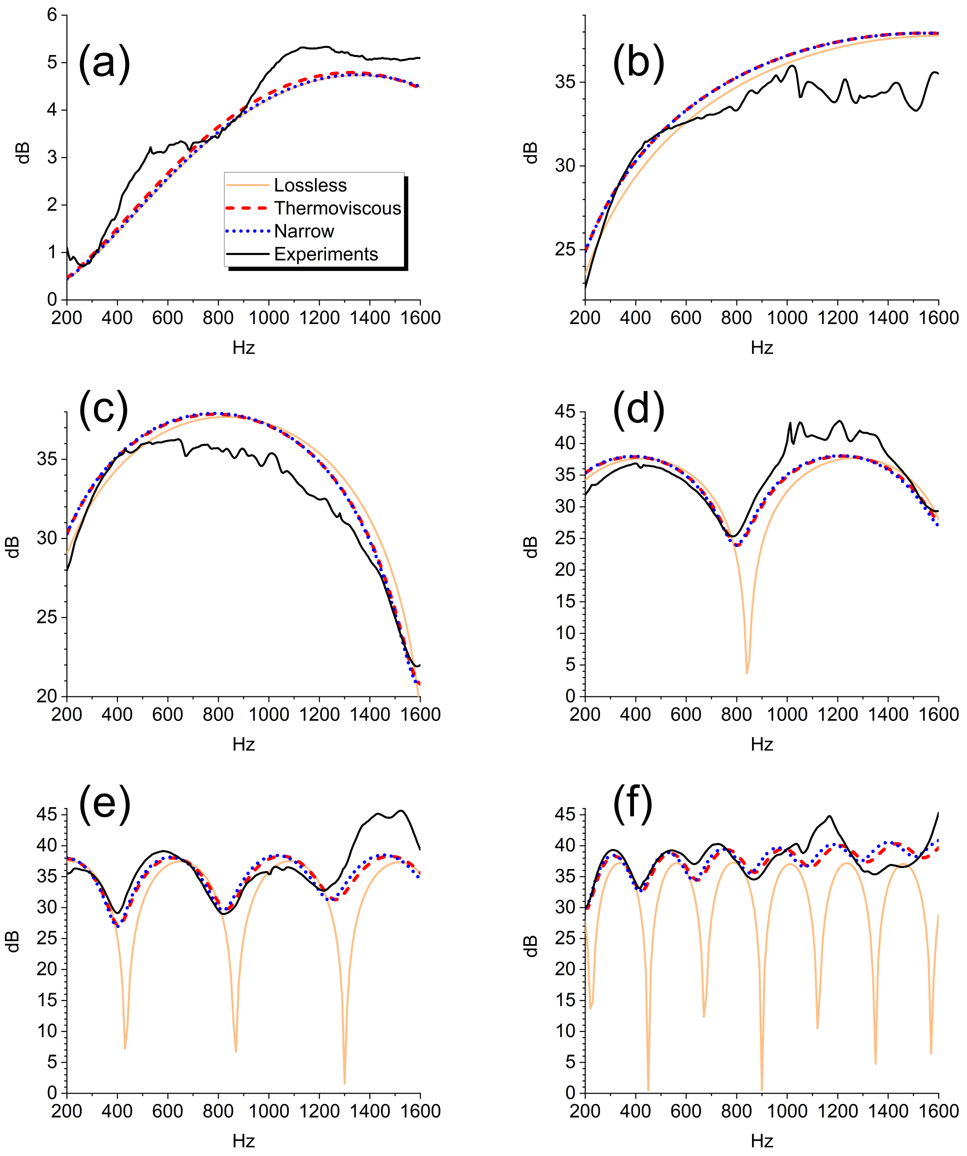

Figure S1. Comparison between results from the experiments and the three FEM models related to the holder and the Hilbert fractals. Fig.S1a, sample holder. Fig.S1b, zeroth order. Fig.S1c, first order. Fig.S1d, second order. Fig.S1e, third order. Fig.S1f, fourth order.

S.2 Comparison between the Hilbert fractal and a bulk block of PLA

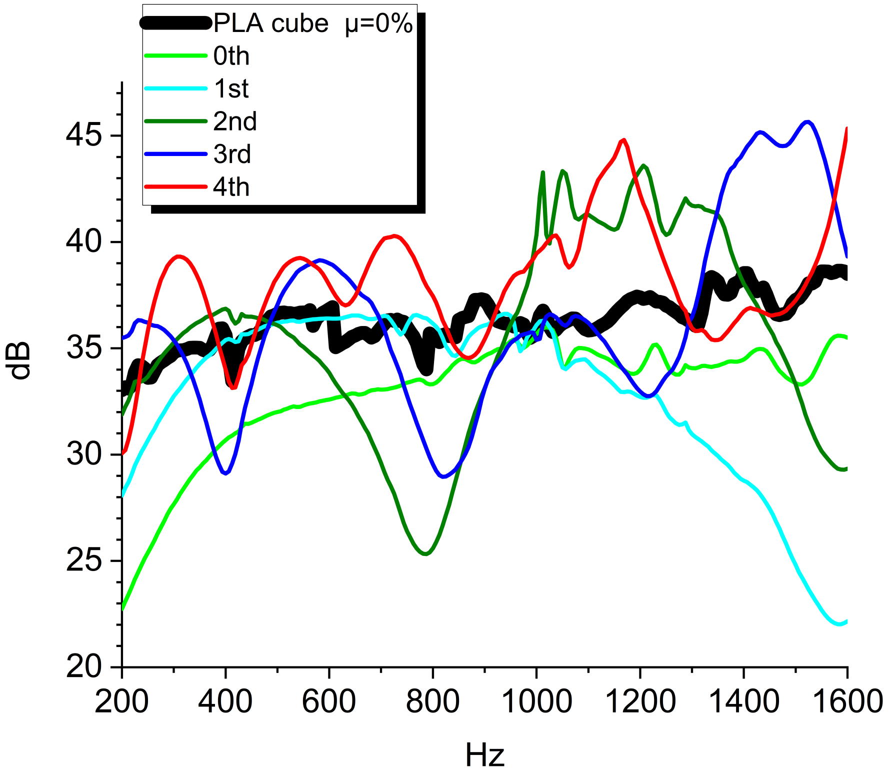

Figure S2. Values of the Transmission Loss provided by Hilbert Fractal Acoustic Metamaterials and a PLA bulk cube. The Hilbert fractal acoustic metamaterials considered here range from 0th order to the 4th order. The PLA bulk cube has a nominal near-zero porosity (i.e., maximum infill ratio). Notably, the transmission losses for Hilbert fractal metamaterials from the 2nd to the 4th order are larger than those of the PLA cube in specific frequency ranges.

S.3 Benchmark of the experimental results shown in this work against those related to other materials and FEM data

We benchmark here our experimental results against those from melamine foam[54] and polyurethane foam[55] panels with the same thickness ($50.8\,mm$), plus three different types of Helmholtz resonators. The first resonator uses an origami approach to modulate the air volume of the resonator chamber[56]. The second one uses a $3\,mm$ internal porous material[57], while the third is a classic resonator[57]. Moreover, we also show a comparison of the FEM transmission losses between the third and fourth Hilbert fractal order, and the transmission loss generated by a multi-slit array with a gap width of $0.2\,mm$, $0.5\,mm$, $1\,mm$, and $2\,mm$. The design of the slits has been taken from the work of *Ward, et al.*[59].

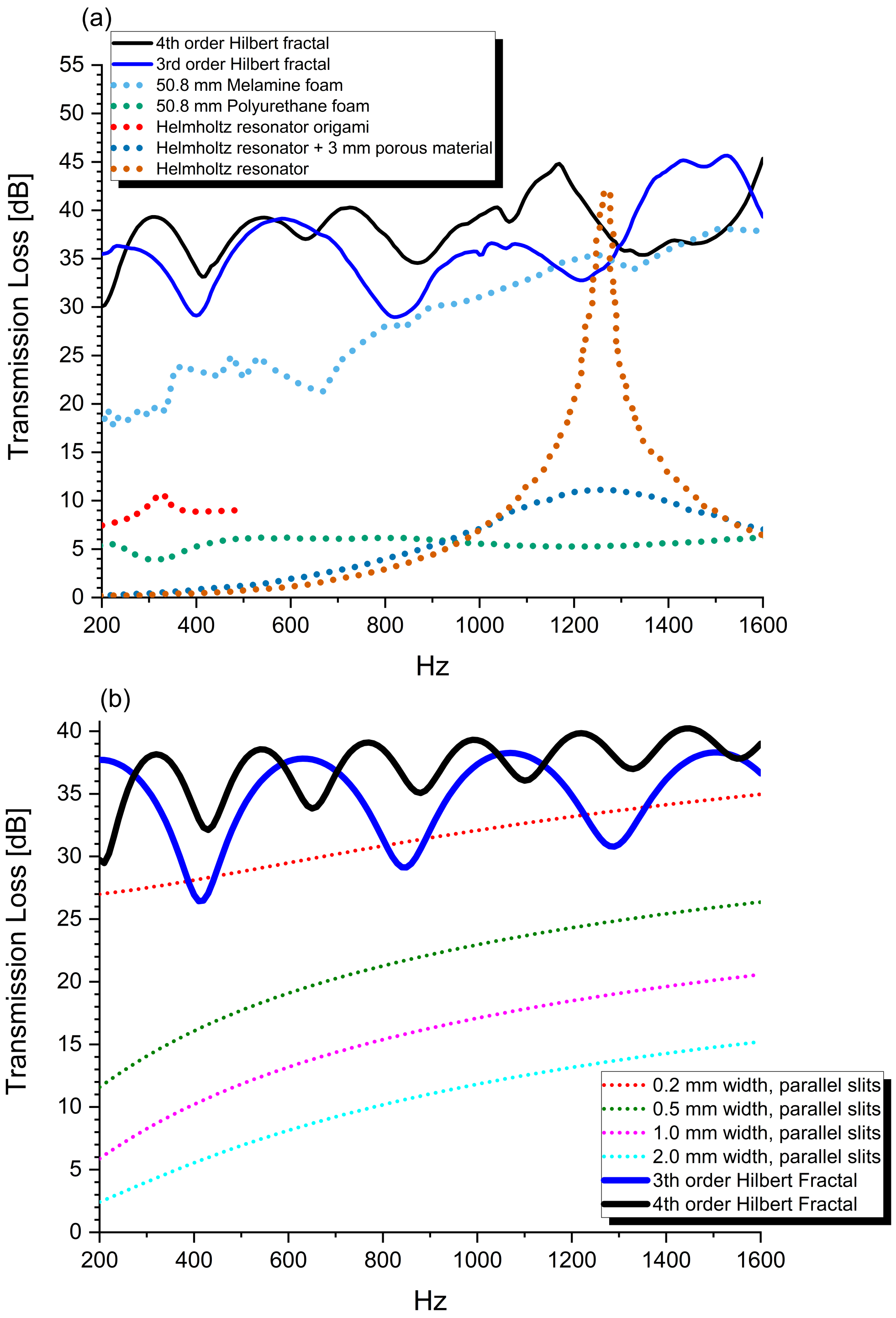

Figure S3. Figure (a) displays the experimental results for Hilbert fractal acoustic metamaterials of orders three and four, melamine foam[54], polyurethane foam[55], an origami Helmholtz resonator[56], a Helmholtz resonator with 3 *mm* of porous material[57], and a standard Helmholtz resonator[57]. All samples, including the foams and Hilbert fractals, have the same thickness of 50.8 *mm*. The Hilbert fractals demonstrate superior transmission loss behaviour in the low-frequency range, while the standard Helmholtz resonator exhibits high transmission losses in a narrowband portion of the spectra. Figure (b) shows FEM simulations related to Hilbert fractal acoustic metamaterials of orders three and four with a 1 *mm* gap width. It also includes the transmission loss related to five straight and parallel slits with gap widths of 2 *mm*, 1 *mm*, 0.5 *mm*, and 0.2 *mm*, based on the geometries described in the work of *Ward, et al.*[59].

S.4 How the fractal acoustic metamaterial generates sound transmission loss

Hilbert fractal acoustic metamaterials operate as pass filters at specific frequencies. As illustrated in Fig.S4, from *(a)* to *(d)*, when a minimum of the reflection coefficient occurs, a reduction in transmission loss happens. A single fractal geometry cavity can have multiple maxima and minima of the impedance mismatch, generating transmission loss peaks according to the overall cavity length, fractal order, side dimensions n of the cube, and gap width w.

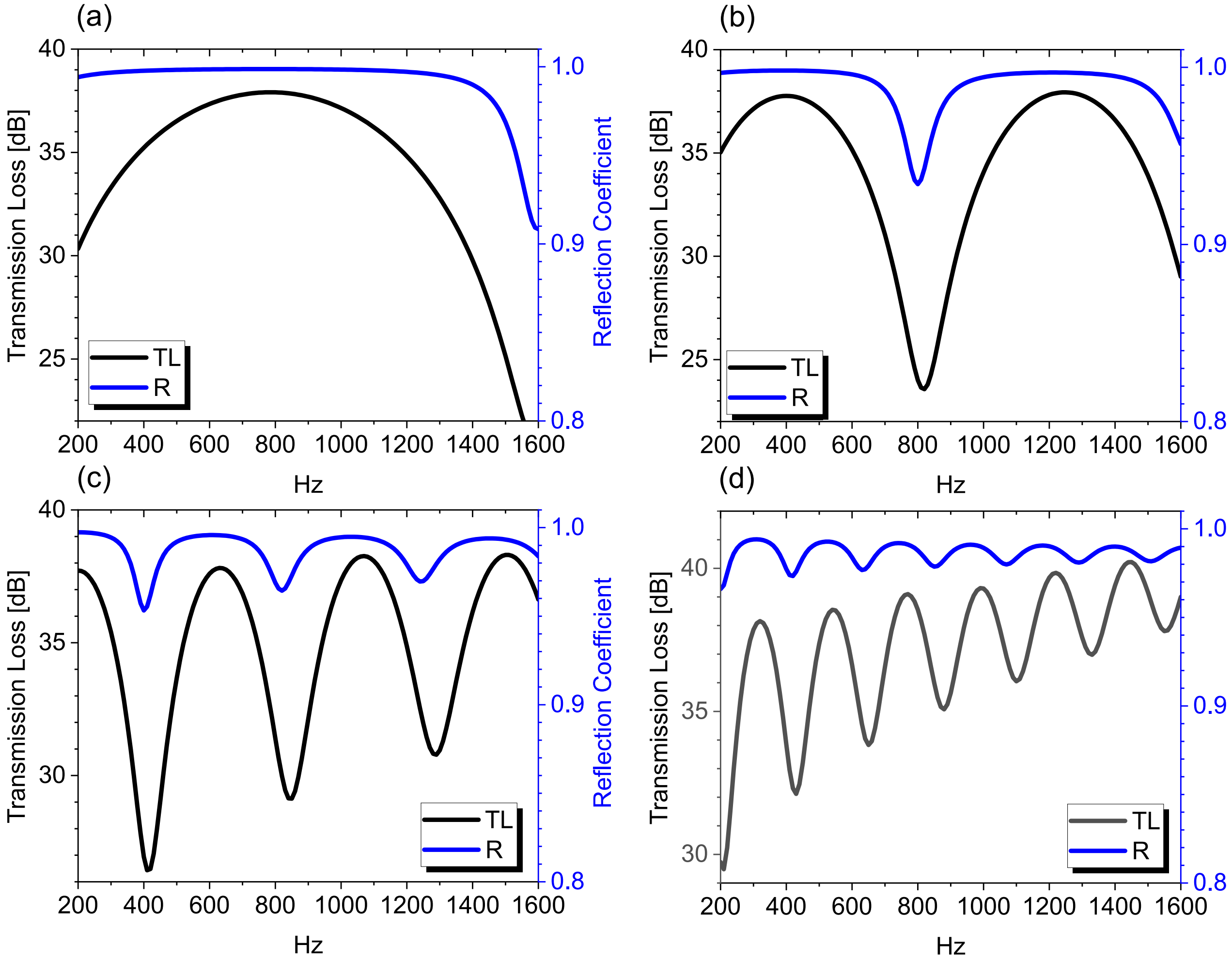

Figure S4. FEM results related to the transmission loss and reflection coefficients for $w = 1\,mm$ gap width. Subfigure (a) presents data related to the first-order Hilbert fractal, (b) for the second, (c) for the third and (d) for the fourth Hilbert fractal orders. Across all subplots, the minima of the reflection coefficient (R) correspond with drops of transmission loss (TL).

S.5 Particle velocity field

To better understand how thermoviscous air dissipation impacts the propagation of acoustic waves within narrow channels, we show the particle velocity field in the lower section of the fractal pattern where the inlet and outlet are positioned (Fig.S5) This plots demonstrate a distinct contrast between the finite element model accounting for the thermoviscous dissipations and the lossless model. This difference becomes particularly apparent as the fractal order increases, and hence the channel length becomes larger. The data clearly show significant discrepancy between predicted and experimented transmission loss occurs when ignoring the effect of the thermoviscous dissipation, as underlined by *Ward, et al.*

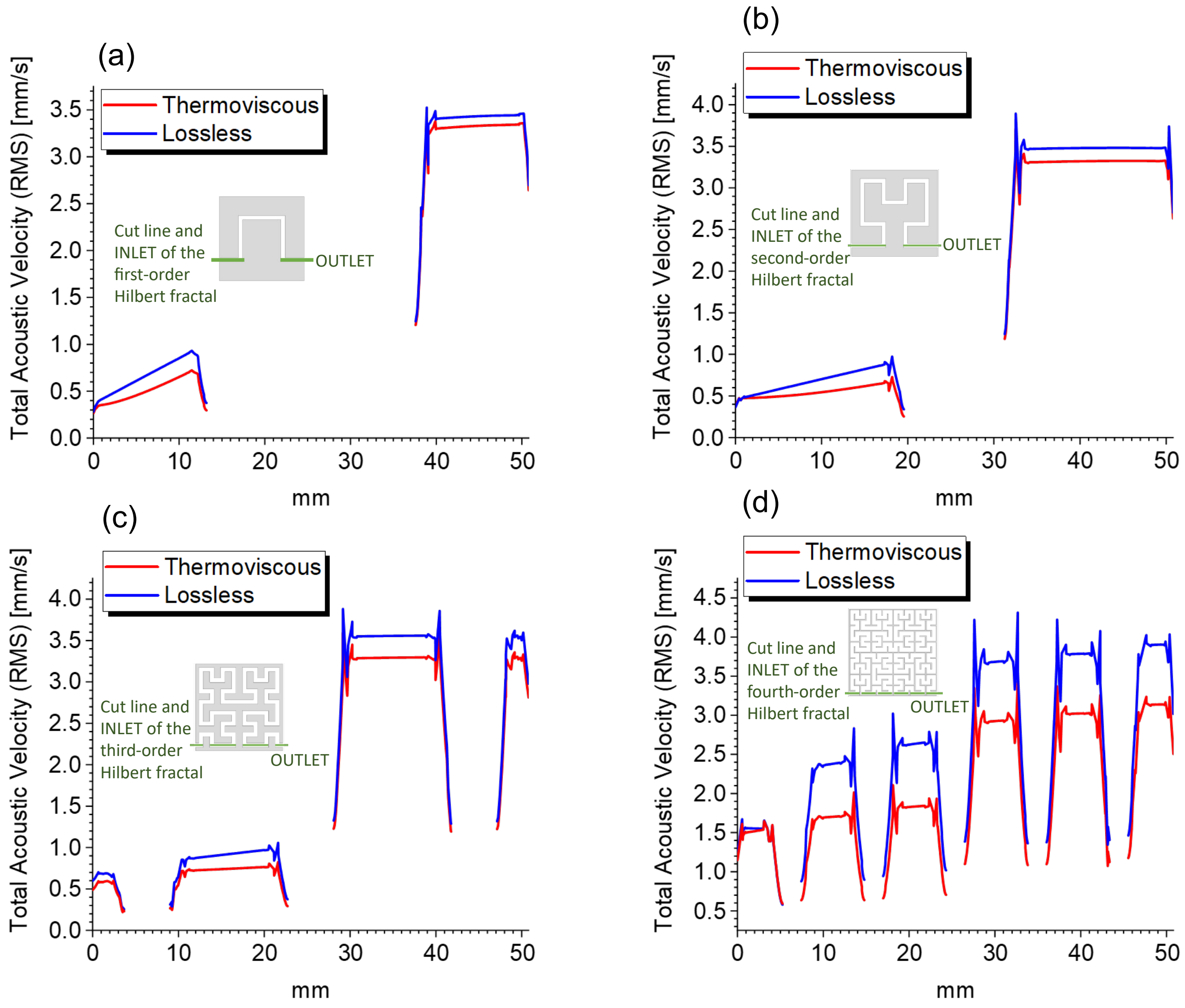

Figure S5. The four subfigures (a, b, c, d) show the total root mean square acoustic velocity in mm/s along a representative line passing through the middle of the inlet and outlet of metamaterials with $w = 1\,mm$ and $n = 50.8\,mm$. Each subfigure presents two FEM simulations: one assuming lossless behavior within the small fractal pattern channels, and the other with a thermoviscous air model. Subfigure (a) illustrates the section line of the first-order Hilbert fractal, represented by a green segment. The absence of a velocity representation in parts of the graph corresponds to the solid portions of the fractal, where air is not present. This pattern is mirrored in subfigures (b), (c), and (d), which are related to the second, third, and fourth-order Hilbert fractals, respectively.

S.6 Normalised standard deviation

Normalised standard deviation for the three different ΔL considered in Eq.1 for every acoustic mode within the frequency range investigated. In the case of $\Delta L = 0$, we observe a fast degeneration of the frequency predicted for acoustic modes after the first. $\Delta L = \frac{8w}{3\pi}$ is the best predictor for the frequencies corresponding to the TL maxima within the gap width range considered in this work. A possible correction for the channel length is $\Delta L = \frac{8}{3\pi^{\frac{3}{2}}}\sqrt{nw}$[59]; when using this expression, one can observe a slightly worse prediction that the one provided by $\Delta L = \frac{8w}{3\pi}$. This is true for all the fractal orders of the metamaterials, apart from the 0th one, for which we have a simple slit without any ninety-degree angles inside. The calculation to normalise the standard deviation is the following:

$$NSD = \frac{A - B}{C} \tag{2}$$

Where NSD is the normalised standard deviation along the several gap widths analysed. A is the average value of the acoustic mode with Eq.1. B and C are the average FEM values for the acoustic modes and for the standard deviation.

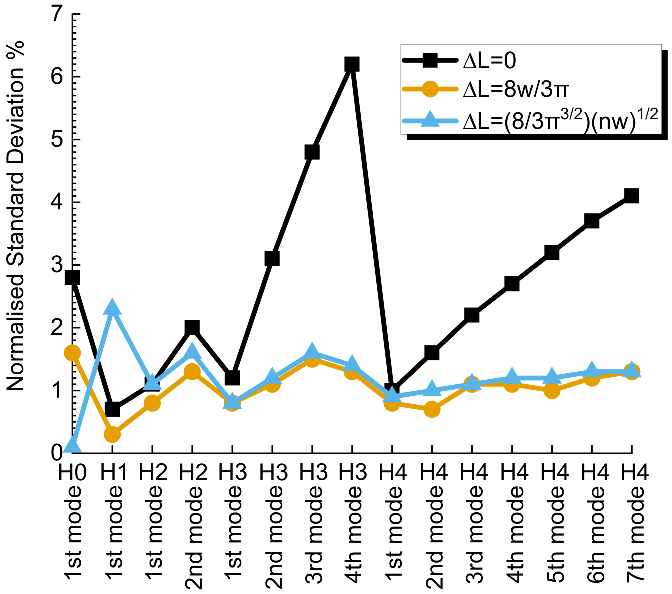

Figure S6. Comparative analysis of the three analytical formulas predicting the frequency position of transmission loss peak between $200\,Hz$ and $1600\,Hz$. The normalised standard deviation is calculated as the difference between the average value of the acoustic modes from the analytical formulas and the FEM simulations, divided by the standard deviation of the frequencies from the FEM simulations. The three formulas differ in considering the correction of the channel length within the fractal. The first formula does not account for any artificial length correction and maintains a reasonably low normalised standard deviation only for the first acoustic mode of the fractals, with an increasing deviation for subsequent modes. The second formula, which considers an artificial elongation as a function of the gap width (w), maintains a low normalised standard deviation across all investigated acoustic modes. The third formula, which considers a length correction as a function of the fractal slit area, also maintains a low normalised standard deviation across all modes but performs worse than the second formula, except for the first acoustic mode of a simple slit without fractal geometry.
